# Pabulum

**Published:** 1914-11

**Authors:** W. Warren Britt

**Affiliations:** Tonawanda, N. Y.


### Pabulum

By W. WARREN BRITT, M. D.

Tonawanda, N. Y.

Pabulum—According to the new Standard Dictionary, is any substance affording nutriment to animals or plants; anything that sustains life and renews organic tissues, as food or drink. Figuratively:—anything that nourishes or develops the mind.

Possibly the history of this club would reveal the fact that the greater part of it's past meetings had been devoted to this subject.

I do not propose to enter upon an elaborate discussion of food values nor methods of its preparation.

We will assume that you all began your career in this world as a healthy, active, crying infant, and that your first introduction to this subject was in the way nature intended it should be.

This paper is not intended to furnish the audience anything in the figurative meaning of the word, but as a matter of interest I have reviewed some literature on infant feeding, which will make one glad he was born in the latter part of the 19th Century.

The mythology and tradition of the beginning of the history of Rome gives us our first example of artificial feeding. Romulus and Remus nursed by the wolf, Romulus becomes the stronger and slays his brother Remus and is thereby the first ruler of Rome.

Children were seldom spoken of in literature before the 18th Century. In certain family letters we find that even high-born mothers expected to lose some of their many children in the perils of teething or weaning, while in medical books there is but one subject spoken of:—namely breast feeding. It was lucky for the infant of the 16th century that there was no question of any other method than the breast of it's own mother or a wet nurse; cow's milk had not been thought of. Until the 15th century, Paulus Fabricius tells us, a child was usually suckled for two or three years, as is common among less civilized people today, but in the days of Thomas Faier, who has many quaint remarks on infant feeding, the time had dwindled to from two years to twenty months, and in spite of Faier's sentiment that it is ''necessary and comely for the owne mother to nourse the owne childe,'' yet there were plenty of mothers who hired a wet nurse to perform their maternal duties. The professional wet nurse was well established by the 16th century, and she maintained her position for 300 years until the 19th century, when the sucking bottle, a sort of pocket wet nurse, was her undoing.

When wet nurses first became the fashion, they were opposed by contemporary moralists as a sign of degeneracy. "It was remembered that Tacitus, just before Rome fell into decay lamented that grave matrons, who in former times used to suckle their own children, now gave them over to Grecian slave girls, and they prophesied an impending judgment on their own race."

But wet nurses flourished notwithstanding, and the physician's lore was devoted to indicating the kind of woman the wet nurse should be. If she were red-haired, evil tempered, or sluttish, the child would suck in vices with his milk. If she ate indiscreetly he would break out in scabbiness or itch.

The mysterious qualities the milk was supposed to possess were plainly set forth by Faier, who says:

"I intend to write somewhat of ye nourse and of ye milke, with ye qualities and complexions of the same. And Phavorinus the Phylosopher (as writeth Aulius Celiues) affirmeth yt if the lambes be nourished with the milke of goates they shall have a coarse wolle, lyke the heare of goats: and if kiddes in like maer suck upo shepe, the heare of them shelbe softe like wolle . . . We see lykewise in herbes and plantes . . . if they be put into unkid earthe or watred with a noughty or unholsome humeur either they come not up at all, or else they will degenerate." Faier logically concluded that lamentable results might follow carelessness in choosing a wet nurse, and if human milk might be potent with moral influences what physiological enormities might come to a human child from drinking the milk of a cow. If a lamb grew a beard from sucking a goat, a child might get a dewlap. Cow's milk was not even discussed in London in Elizabeth's day.

Had it theoretically been considered a suitable food it could hardly have been used practically, for although there were pastures within ten minutes of most dwellings in London, yet the unsanitary conditions of the town would have made it impossible for a child to have lived upon milk.

"London" says Dr. Forsythe, in his article before the Royal Society of Medicine, "then stretched from Ludgate to the Tower and from London Wall to the Thames. This small area was covered with a meshwork of narrow lanes and streets, across which the projecting upper stories of the wooden houses nearly met. Roadways and paths were hardly kept, while haystalls and stinking heaps of refuse encumbered every quarter. There were latrines but no drains. At the back of every house stood a cesspool! Amid these surroundings lived a population which even before the sixteenth century had become too numerous for the city, but was prevented from spreading beyond its walls by the ecclesiastical owners of the surrounding manors. To make matters worse, Elizabeth who had no wish to see London larger, prohibited any new buildings within the walls. Overcrowding was the result, and from a royal proclamation of 1580 we learn that in the poorer quarters there were "great numbers of people inhabiting in small rooms."

The annual bills of mortality were first published by Elizabeth, in the hope, it is said, that by making known risks of living in London, intending residents would stay away. Within doors the sanitary conditions were no better than in the streets. Writing to Travers, the physician to Cardinal Wolsey, Frasmus describes the filthy state of the dwelling houses. The floors, he says, were commonly of clay, and were strewn with rushes, beneath which lay, unmolested for years, an ancient collection of spittle, beer, decayed fish and other refuse even more disgusting.

Filthy streets, overcrowded houses, decaying refuse in the living rooms,—these were the conditions that made artificial feeding impossible.

Further—we must remember that the virtue of boiling the cow's milk was not only unknown among all classes, but was even denied by the faculty. Popular prejudice indeed held that boiled milk was actually injurious, a belief that has not been eradicated even in our own generation. To complete the picture we must add that bodily cleanliness was the lot of but few babies, even of the highest in the land. Parents who rarely if ever, took a bath, were not likely to attach much importance to washing their children; besides what nurse could be bothered to unswaddle a babe—stays, swathes, rollers, fillets and bands, every time it needed attention.

Hand feeding for babes under these conditions would have meant certain death, and fortunately, the wet nurse was the only alternative for the mother's breast. London grew still more unhealthy, although in the days of James, the city burst through the gates and built along the river, and smallpox, plague and the sweating sickness worked their ravages. By the time of the Restoration, the infant mortality was terrible. Two-fifths of the total deaths were of children under two years. In some years more than half the births were wiped out by infantile disease. When a hot summer came and the stench of the offal rose up off the streets, the children died like the flies that swarmed in the overcrowded tenements. We read how in each of the hot summers, 1669, 1670 and 1671, diarrhea alone added two thousand to the bills of mortality in eight or ten weeks.

It is not surprising, therefore, that as late as 1679, there is no mention of artificial feeding in the "General Treatise of the Disease of Infants and Children,'' published by John Peachy. He still advises a ''merry'' wet nurse. He shortens, however, the period of weaning, and says ''a child should suck a year and a half to two years,'' but his golden rule is ''The Child must not be weaned until it has all it's teeth.''

''Wise people held, and not without a reason,'' says Peachy, ''that a child must not be weaned with a moon on the wane. The first additional food should be ''pap'' and chicken broth, and bread and milk.'' Juicy meat he allows about the same time, but with the homely recommendation, that it be ''first chewed by the nurse.''

In the 18th century nobody improved on Peachy's rules, though the period of weaning was shortened 18 to 20 months, with the solemn warning, however, that ''if the child has not cut his teeth even at the end of two years, and is nevertheless weaned, he will run a great risque of his life.''

But the increasing demand for wet nurses was followed by a new evil. The child of the nurse, having to be brought up by hand, usually died. No notice was taken of this state of affairs until two philanthropic medical men, agitated the subject and urged wealthy families to open an institution where the children of the wet nurses could be looked after. But this remedy only touched a few. Many wet nurses did not care to have their children saved. It became the fashion, during the 18th century to choose a young, healthy girl who had had an illegitimate child, preferably her first one, in preference to a married woman. The work of a wet nurse was light, much easier than that of a servant, the wages better, ten to twelve shillings a week, with tea and sugar included—and the position in a household pleasant; therefore it was a very desirable thing to have an illegitimate child, especially as when it was put out to a baby farm it never lived long. As the evil progressed, it was found still simpler to have the child stillborn.

And so in the 18th and the beginning of the 19th century, the wet nurse reigned supreme. When artificial feeding was introduced, her calling began to waver, and when a clean feeding bottle was invented, it received it's death blow.

In the middle of the 18th century we read that ''when a child sickens it is usual to feed it with nothing but water pap—that is, bread and water boiled together.'' Mothers generally nursed their children until they were two years old, but gave them water pap along with breast milk, any time after cutting the first tooth. Even if no breast milk were available it was thought safer to give water altogether instead of cow's milk.

Later the pap began to be made of ''baked flour'' and as both the paps proved so successful, it became the custom to make chicken broth, or beef tea, and Lisbon sugar was added to the babies' hitherto meager menu.

Dr. Michael Underwood, in the early part of the 19th century, who it may be remembered, gave one of the earliest descriptions of poliomyelitis, was the first to analyze various milks and to recommend the use of cow's milk for the average child or asses' milk for tender infants, or when cow's milk set up purging.

He had the insight to perceive that the real failure in hand feeding, was due to the perishable quality of milk, and urged that it be boiled and diluted with barley water. He also advocated rice which was then new in England, and tapioca and semolina. He reduced the time of nursing to 12 months, but insisted that at least four teeth be cut.

A cow's horn was the first sucking bottle. It was taken from a small cow or calf, scraped and polished, and it's tip perforated, while around this hole were sewed two small pieces of parchment or leather, like the finger of a glove. It was soon perceived that any child brought up on it was "in danger of falling into watery gripes," and small wonder with the accumulation of soured milk in the tip of the horn and leather.

Before long, a glass bottle was introduced, shaped like a horn, and a pap boat, called pap spoon, with a hinged lid, was tried. The glass bottle, though cleaner than the horn, was rendered as harmful by the various kinds of teats—leather and parchment, stuffed with a sponge, decalcified bone teats, wooden teats, and India rubber teats (which, however, had in earlier days, a "repulsive taste and smell"). One of the most popular was a heifer's teat, which "was prepared in the best possible manner by those that understood the art," and tied to the bottle with a thread. It had to be kept in spirits to keep it from putrefying, and the sign that a new one was necessary was the child's refusal to take it between it's lips.

France contributed a very wonderful feeding bottle called the "biberon," with glass tubes to conduct the milk and let air in and also a receptacle with a large, breast-shaped nipple called the "mamma," but the moment the relation of germs to food was understood and the necessity for cleanliness made plain, all tubes of rubber or glass and fancy bottles were thrown aside, and the simple bottle and the small, washable, elastic rubber nipple came into use.

Until the clean feeding bottle was introduced, we find even as late as 1867, authorities urging the direct sucking of cow or ass, rather than any form of hand feeding. This fanciful advice given to Englishmen is explained by the disrepute in which milk was justly held, especially in London where for lack of transit facilities dairymen stalled their cattle in various parts of the city, as was the case in New York thirty years ago.

The Lancet appointed a sanitary commission to investigate the condition of these houses, and records as "by no means an exceptional case 39 to 40 cows in a most disgusting condition, full of ulcers, their teats diseased, and their legs full of tumors and abscesses."

And in a pamphlet entitled "Observations on London Milk," 1851, the following passage occurs:

"There is scarcely a cowshed one enters but some of the beasts will be found afflicted with mange. In some sheds, the poor beasts never saw the light of heaven from year's end to year's end."

Naturally, writes Dr. Forsythe, with these conditions some safer food than cow's milk was pressing. Sago milk, arrowroot, salep, biscuits, "tops and bottoms," gum arabic, manna-croup, isinglass and jellies were tried, but many of these had but short vogue, their disappearance being hastened by the appearance of the first proprietary foods, which were placed on the market in 1840.

It was Dr. Golding, the founder of Charing Cross Hospital, who realizing the need of a cheap, but safe, food for the poor, induced a London firm to specially prepare the so-called "tops and bottoms" for the general public. It met with such immediate success that other prepared foods soon followed. In 1867, Justus von Liebig introduced a food in the preparation of which he introduced the principles of malting, but it had the inconvenience of having to be supplied fresh daily; but although in itself, was not wholly successful, the principle of it's preparation was good, and has survived in many foods today.

The next step was the introduction of concentrated, condensed or dessicated cow's milk. This improvement gave a considerable impulse to the practice of artificial feeding, and to the manufacture of patent foods. So that by 1883, no fewer than 27 brands could be obtained. Many of these required the addition of milk, which rendered them open to the dangers of milk diet.

"And by the middle of the 19th century," he continues, we find the hand fed child at last provided with a fairly satisfactory feeding bottle, animal milk, which was none too clean, and some choice in proprietary foods.

The next point to which physicians turned their attention was the dilution of the milk and foods and the quantity to be fed. It was Dr. Eustace Smith, who by moderate and intelligent advice, made it possible in the latter half of the 19th century to write for the first time:—"The successful rearing of an infant by artificial means is not a difficult matter. We find also, that infants cannot be fed only by schedule made out for relative ages, their weights vary and they require different amounts of nourishment, so that in place of age, we must look for a physiological basis and understand the infant's requirements of animal heat.

Another likely development is in the use of foods other than milk. Human milk has long been increasingly supplanted by cow's milk. Cow's milk in its turn, is meeting with the rivalry of patent foods. Cow's milk is open to obvious and serious objections. It is easily contaminated. It is frequently diseased. It is both frequently and easily tampered with before it reaches the infant. These disadvantages are not readily discovered, except by the harm they produce. At best, cow's milk is variable in composition, bulky in transit, inconvenient to store, and soon loses its sweetness.

The patent foods on the other hand, with which it has to contend, are prepared by scientific methods that prevent the risk of contamination. They are never diseased, rarely tampered with and usually are consistent in composition. Prepared in a concentrated, solid form, they are most convenient in transit and storage, they retain their freshness, and are readily converted into a liquid such as the infant requires.

On the whole, the future of infant feeding, must take advantage of artificial means.

We have all tried to carry out the principle of ''Mother's milk for every baby.'' We try to teach mothers its importance. But we do meet cases where artificial food must be introduced.

There is no Best Brand of Artificial Food. Each case is a law unto itself. We must study cause and effect.

The burden of carrying the message of clean milk, and methods of preparing artificial food to the mothers of the land, has been upon the medical profession. Too often the message is not heeded until too late.

But with the establishment of Child Welfare Stations throughout the State and the practical demonstrations to young mothers, may we not see an awakened public opinion and the observance of better hygiene and sanitation throughout our common wealth?

No. 32 Grove Street.

---

**Joseph Popper,** in the Dietetic and Hygienic Gazette, June, 1914, gives a very good and readable survey of the main points on ''The feeding of Infancy and Childhood'' which is intended mainly for the instruction of mothers, but which is well worth the perusal by the general practitioner. Papers of this nature are of great importance and they are sure to accomplish a great deal of good.—C. G. L-W.

